# Supplementary material for: Mechanical Load and Piezo1 Channel Regulated Myosin II Activity in Mouse Lenses
Source: Int J Mol Sci. 2022 Apr 24;23(9):4710. doi: 10.3390/ijms23094710 (PMC9105872; doi:10.3390/ijms23094710)
Supplement: Supplementary file 1 [file ijms-23-04710-s001.zip › ijms-1650903-supplementary.pdf]

Mechanical Load and Piezo1 Channel Regulated Myosin II Activity in the Mouse Lens

Ariana Allen [1] Rupalatha Maddala [1] Camelia Eldawy [1] and Ponugoti Vasantha Rao [1, 2] *

1 Department of Ophthalmology, Duke University School of Medicine, Durham, NC 27710, USA

2 Department of Pharmacology and Cancer Biology, Duke University School of Medicine, Durham, NC 27710, USA

* Correspondence: p.rao@duke.edu

Supplemental Material:

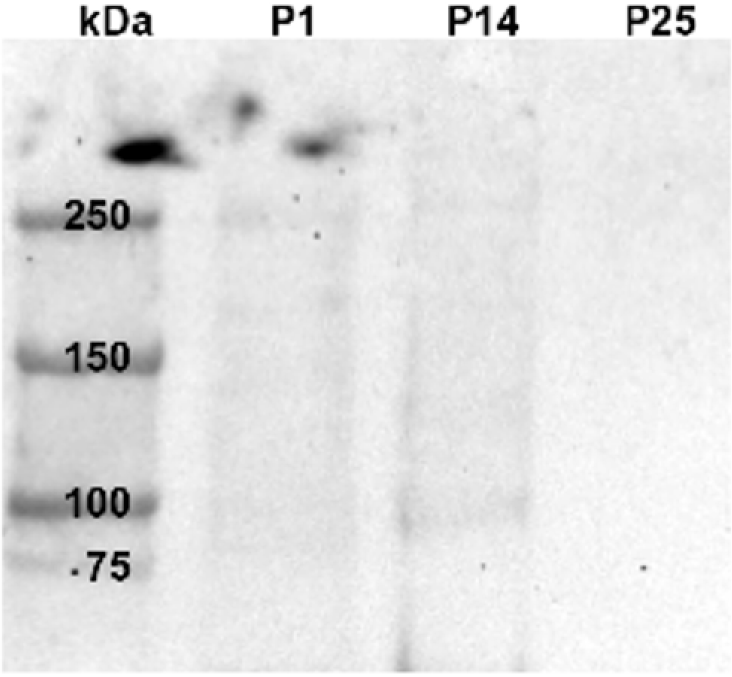

Figure S1. Immunoblot analysis of Piezo2 protein in the mouse lens lysates.

To detect Piezo2 protein in lens, the total lysates prepared from the P1, P14 and P25 mouse lenses (75 µg protein) were subjected to immunoblot analysis, and Piezo2 was not found to be detectable.

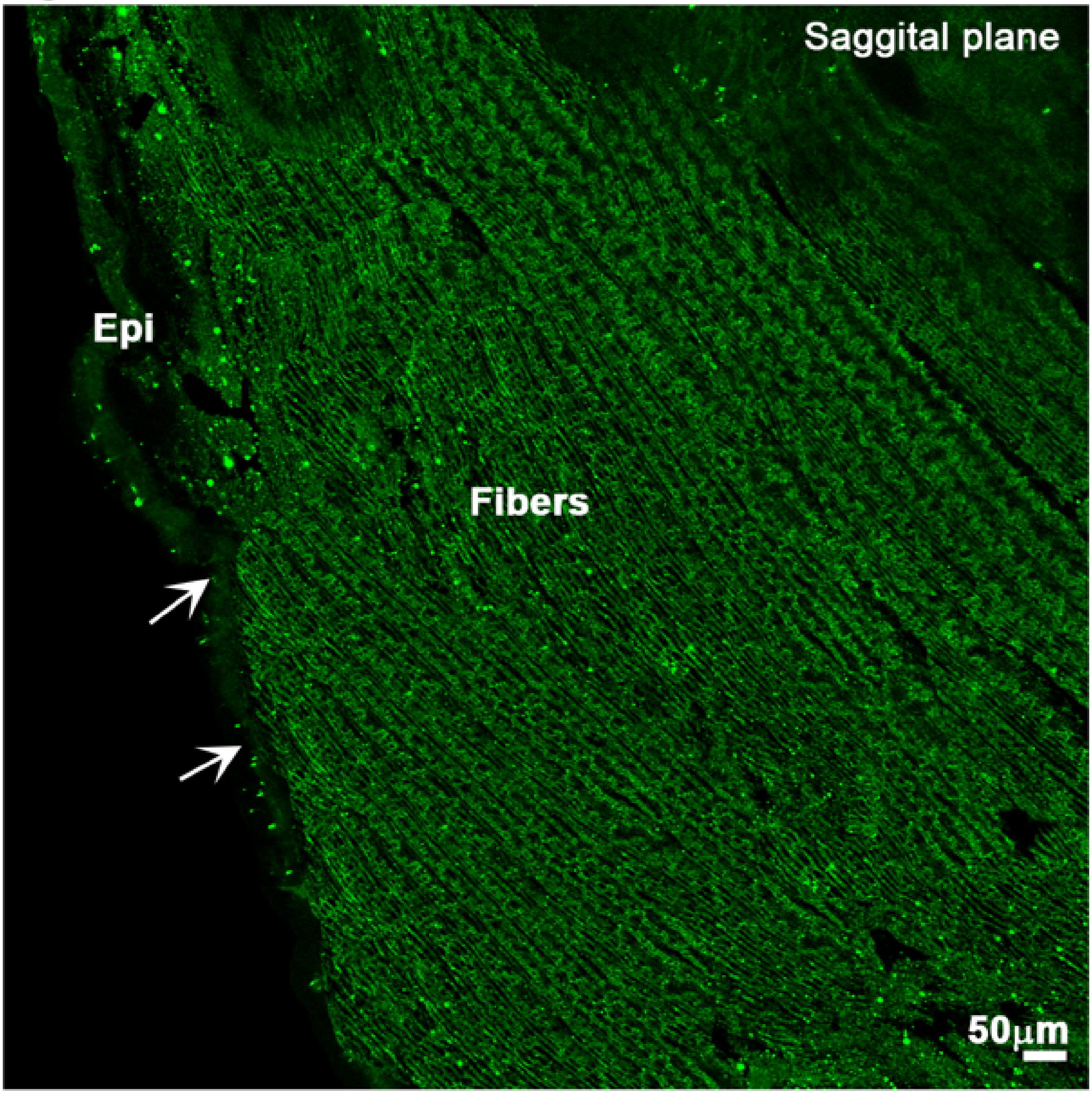

Figure S2: Piezo1 distribution in the epithelium of mouse lens.

While the sagittal sections of Piezo1^{-tdT} mouse lens immunostained against the tdT protein reveal an intense distribution of Piezo1-tdT fusion protein (bright green fluorescence) in the lens fibers, it is barely detectable in the lens epithelium (arrows indicate the area where the epithelium is intact with fibers). Scale bar indicates image magnification.
